# Molecular subtypes and prognosis in RCC

**DOI:** 10.18632/aging.100736

**Published:** 2015-04-10

**Authors:** Benoit Beuselinck, Wolf Herman Fridman, Jessica Zucman-Rossi

**Affiliations:** UMR-1162, Génomique Fonctionnelle des Tumeurs Solides, IUH, F-75010 Paris, France

Oncologists are facing several major challenges including growing cancer incidence in an aging population, and increasing costs of new anti-cancer drugs. A better understanding of tumor biology and heterogeneity should lead to the development of new predictive biomarkers allowing to tailor therapies in order to increase therapeutic efficacy and more rationally use the health care budget.

Since 2005, anti-vascular endothelial growth factor receptor targeted tyrosine kinase inhibitors (anti-VEGFR-TKIs) such as sunitinib and pazopanib have significantly improved the prognosis of metastatic clear-cell renal cell carcinoma (m-ccRCC)-patients. Nevertheless, selecting patients who might benefit from these therapies remains a challenge since no reliable biomarkers of sunitinib sensitivity or primary/secondary resistance have been identified [1].

Recently, through an unsupervised transcriptome analysis, we discovered a molecular classification of ccRCC with four robust subgroups (ccrcc1 to 4) related to previous molecular classifications [2, 3] and associated with outcome on sunitinib [4]. Non-responders (progressive disease as best response) were enriched in ccrcc1 (22%) and ccrcc4 (27%) versus 3% in ccrcc2 and 0% in ccrcc3. In contrast, partial or complete responses were over-represented in ccrcc2 (53%) and ccrcc3 (70%) compared to 41% and 21% in ccrcc1 and ccrcc4, respectively (p=0.005). Accordingly, ccrcc1 and ccrcc4-tumors when compared to ccrcc2 and ccrcc3-tumors showed a poorer median progression-free survival (mPFS) (13, 8 versus 19 and 24 months, respectively; p=0.0003) and median overall survival (mOS) (24, 14 versus 35 and 50 months, respectively; p=0.001). Classification of the tumors belonging to ccrcc1&4 versus ccrcc2&3 subgroups was the most significant covariate in univariate cox analysis with a poorer mPFS (p=0.017) and mOS (p=0.006).

We further characterized these four molecular subgroups. Whereas mutations in *VHL* and *PBRM1* were rarely found in ccrcc4-tumors, they were frequent in ccrcc1- and ccrcc2-tumors but without relationship with sunitinib response. ccrcc1/ccrcc4-tumors were characterized by a stem-cell polycomb signature and CpG hypermethylation. ccrcc4-tumors exhibited frequent sarcomatoid differentiation with a strong inflammatory, Th1-oriented but suppressive immune microenvironment, with high expression of *PDCD1 (PD-1)* and its ligands *PD-L1* and *PD-L2*. ccrcc4-tumors showed several characteristic copy number aberrations, the most significant being 2p12-, 2p22.3- and 8q21.13-amplifications. Both ccrcc1- and ccrcc4-subtypes over-expressed *MYC*-targets. The expression and methylation profile of ccrcc3-samples was similar to that of normal kidney tissue concerning metabolic pathways and transporter activities. The ccrcc2-subtype was not characterized by specific pathways; it always showed an intermediate expression signature, comprised between ccrcc3 and (ccrcc1/ccrcc4) related profiles. ccrcc2-tumors showed the highest mutation rate for *VHL*. In ccrcc2-tumors, the “cellular response to hypoxia” pathway was less activated than in the ccrcc1/ccrcc4-subtypes, whereas ccrcc3-tumors did not exhibit cellular response to hypoxia. Based on these molecular characteristics, we renamed our subtypes as follows: ccrcc1=“c-myc-up”, ccrcc2=“classical”, ccrcc3=“normal-like” and ccrcc4=“c-myc-up and immune-up”.

Association of this novel renal cancer molecular classification with response to sunitinib and survival remains to be evaluated in patients treated for metastatic clear-cell renal cell carcinoma with other anti-VEGFR-TKIs. Recently, we obtained very promising results in a series of 16 tumors of patients treated at the University Hospitals Leuven with pazopanib (unpublished results). In this small series of patients, mOS is significantly shorter in ccrcc-1 and ccrcc4 tumors compared to ccrcc2/ccrcc3-tumors (p<0.0001).

These findings allow us to hypothesize on the working mechanism of anti-VEGFR-TKIs in m-ccRCCs. The best responding ccRCCs seem to be those in which hypoxia pathways are not (ccrcc3) or less (ccrcc2) activated compared to resistant ccrcc1/ccrcc4-tumors. Moreover, patients with initially hypoxic targets, as assessed by a PET-CT-scan with 18F-fluoro-misonidazole, have a shorter PFS on sunitinib than patients with non-hypoxic targets [5]. Hypoxia is associated with tumor aggressiveness through higher HIF-levels and expression of genes involved in tumor proliferation, vasculature, invasion, and metastatic spread leading to a poor prognosis. Through the reduction and normalization of blood vessels, anti-VEGFR-TKIs leads to better oxygen delivery in the tumor, lowering hypoxia. In most tumor types, neoangiogenesis is triggered by hypoxia. Nevertheless, in ccRCCs, particularly hypervascular tumors, the dysregulation of the VHL-protein plays an important role in pathogenesis and neoangiogenesis. Thus, an attractive hypothesis is that the balance of neoangiogenesis versus hypoxia is a major trigger of response to anti-VEGFR-TKIs. Therefore, in opposition to other tumor types, anti-VEGFR-TKIs can work as a monotherapy in ccRCCs, at least in a subgroup of ccRCCs with low hypoxia. On the opposite, in fast growing ccRCCs, for instance due to 8q21- and c-myc-amplification, hypoxia will be more important leading to an unfavorable neoangiogenesis/hypoxia balance. The question remains if and how resistance could be countered improving the neoangiogenesis/hypoxia balance.

In the new era of developing clinical trials guided by molecular features of the tumors, our molecular classification could be used to stratify patients that could benefit from anti-VEGFR-TKIs treatments. Moreover, we can reasonably hypothesize that in patients with ccrcc4-tumors, hypomethylating agents targeting epigenetic defects [6] or immune-modulatory antibodies [7] should be preferentially tested. Further validation of these findings is warranted in future clinical trials integrating molecular subtyping in their design.
